# Association Between Nutrient Patterns and Fatty Liver Index: Baseline Survey of the Japan Multi-Institutional Collaborative Cohort Study in Tokushima, Japan

**DOI:** 10.2188/jea.JE20200447

**Published:** 2022-08-05

**Authors:** Nguyen Van Tien, Kokichi Arisawa, Hirokazu Uemura, Nahomi Imaeda, Chiho Goto, Sakurako Katsuura-Kamano

**Affiliations:** 1 Department of Preventive Medicine, Tokushima University Graduate School of Biomedical Sciences, Tokushima, Japan; 2 Department of Health and Welfare System, College of Nursing Art and Science, University of Hyogo, Hyogo, Japan; 3 Department of Nutrition, Faculty of Wellness, Shigakkan University, Aichi, Japan; 4 Department of Health and Nutrition, Nagoya Bunri University, Aichi, Japan

**Keywords:** fatty liver index, nutrient patterns, cross-sectional study, factor analysis

## Abstract

**Backgrounds:**

The fatty liver index (FLI) is a good non-invasive approach for fatty liver disease diagnosis. The objective of this study was to examine the associations of nutrient patterns with non-alcoholic fatty liver disease (NAFLD) in a Japanese population.

**Methods:**

A total of 1,588 subjects (789 men and 799 women) aged 35–69 years were recruited in the baseline survey of the Japan Multi-Institutional Collaborative Cohort (J-MICC) Study in Tokushima Prefecture. Factor analysis was applied to energy-adjusted intake of 21 nutrients, and nutrient patterns were extracted. Multiple logistic regression analysis was used to analyze the relationships between nutrient patterns and the high FLI category (≥60).

**Results:**

Four nutrient patterns were extracted: Factor 1, vitamins, dietary fiber, iron and potassium pattern; Factor 2, fats and fat-soluble vitamins pattern; Factor 3, saturated fat, calcium, vitamin B_2_ and low carbohydrate pattern; and Factor 4, sodium, protein and vitamin D pattern. After adjustment for sex, age, and other potential confounding variables, higher Factor 1 scores were significantly associated with lower odds ratios of NAFLD (*P* for trend <0.05). Analysis of each component of FLI showed that there were significant inverse associations between Factor 1 scores and high body mass index and large waist circumference.

**Conclusion:**

The present findings suggest that a nutrient pattern rich in vitamins, fiber, iron, and potassium was associated with lower prevalence of NAFLD in a Japanese population. Obesity and abdominal obesity may be intermediate variables for the association between this nutrient pattern and NAFLD.

## INTRODUCTION

Non-alcoholic fatty liver disease (NAFLD) is one of the most common chronic liver diseases, characterized by an excessive hepatic fat content above 5%, without evidence of heavy alcohol intake (>30 g/day for men, >20 g/day for women) or use of drugs.^1^^–^^3^ NAFLD often coexists with obesity, dyslipidemia, insulin resistance, and type 2 diabetes mellitus, and patients with NAFLD are at increased risk for future development of type 2 diabetes, cardiovascular diseases, liver cirrhosis, and hepatocellular carcinoma.^4^^–^^7^ Therefore, early detection of NAFLD and improvements in lifestyle are crucial to prevent more severe health consequences. Although liver biopsy is considered to be the gold standard for the diagnosis and staging of fatty liver disease, Bedogni et al reported that the fatty liver index (FLI) was a simple and non-invasive scale to diagnose fatty liver disease.^8^ In recent years, several studies have proved the usefulness of the FLI for the diagnosis of NAFLD in epidemiologic studies.^9^^–^^11^

Dietary pattern analysis has been frequently used to investigate the association of consumed foods or food groups as a whole and the associated risk of chronic diseases. It extracts factors that explain the total variance in food intake among individuals as much as possible using statistical methods, such as factor analysis. The relationship between dietary patterns and NAFLD has been reported in some previous studies. For instance, studies in China showed that dietary patterns, characterized by high intake of coarse grains, tubers, vegetables, and beans, were inversely associated with prevalence of NAFLD.^12^^,^^13^ In contrast, consumers preferring the traditional Korean dietary pattern, which was characterized by high intake of vegetables, fermented vegetables, fish and seafood, mushrooms, and soybeans, exhibited a higher prevalence of NAFLD.^14^ In Australia, a Western dietary pattern characterized by high intake of red meats, processed meats, full-fat dairy products, fried potatoes, refined cereals, cakes and biscuits, confectionery, and soft drinks, was associated with a higher risk for developing NAFLD.^15^ Some studies showed that people following the Mediterranean dietary pattern, rich in olive oil, fish, nuts, whole grains, fruits, and vegetables, were at reduced risk of NAFLD.^16^^,^^17^

Recently, nutrient pattern analysis has been applied to examine the association between overall nutrient intake and chronic diseases. This method extracts factors from nutrient intake data instead of food intake. The advantages of nutrient pattern analysis are that it helps to look at the combination of bioactive nutrients in complex biological mechanisms, and it is easier to apply to international studies.^18^^,^^19^ To our knowledge, there have been no previous studies that examined the association between nutrient patterns and NAFLD. The purpose of the present study was to examine the nutrient patterns associated with high FLI in a Japanese population.

## METHODS

### Study subjects

The Japan Multi-Institutional Collaborative Cohort (J-MICC) Study was started in 2005, and its main purpose was to detect and confirm environmental factors and genetic traits associated with lifestyle-related diseases, including cancer, among Japanese.^20^ In Tokushima Prefecture, we recruited 2,440 subjects (1,414 men and 1,026 women) aged 35–69 years from three groups, between January 2008 and February 2013. The first group consisted of 570 participants who received health check-ups at the Tokushima Prefectural General Health Check-up Center. The second group comprised 1,174 employees of companies located in Tokushima Prefecture. The third group consisted of 696 subjects who voluntarily participated in this study after reading leaflets which were distributed to each household in Tokushima city.

**Figure 1.**
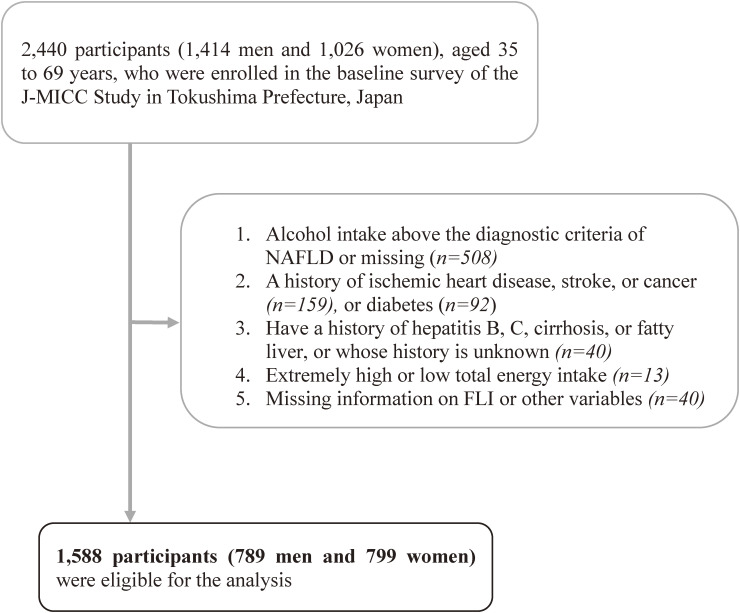
A flowchart showing the process of selecting study subjects. J-MICC, Japan Multi-Institutional Collaborative Cohort; NAFLD, non-alcoholic fatty liver disease.

We excluded participants who drank alcohol above the diagnostic criteria for NAFLD (male >30 g/day; female >20 g/day) (*n* = 508); who had a previous history of ischemic heart disease, stroke, or cancer (*n* = 159); who had diabetes (*n* = 92); or who had a history of hepatitis B, hepatitis C, liver cirrhosis, or whose disease history was unknown (*n* = 40). We also excluded study subjects with extremely high or low total energy intake (≥4,000 kcal/day or <1,000 kcal/day) (*n* = 13) and participants who lacked either the height, weight, serum triglycerides (TG), γ-glutamyl transferase (GGT), waist circumference (WC) necessary for FLI calculation, and other variables (*n* = 40). Finally, 1,588 participants (789 men and 799 women) were eligible for the present data analysis (Figure 1).

### Questionnaire

We used a structured, self-administered questionnaire to collect personal information on lifestyle. To evaluate the subjects’ dietary habits, food consumption data were collected from a food frequency questionnaire (FFQ).^21^^–^^24^ The subjects were asked to answer to questions about intake frequency of 46 items of foods and beverages in the past year. The frequency of consumption of rice, bread, and noodles at breakfast, lunch, and dinner were divided into six levels: rarely, 1–3 times/month, 1–2 times/week, 3–4 times/week, 5–6 times/week, and every day. For other food items, including coffee and green tea, frequency was divided into eight categories: rarely, 1–3 times/month, 1–2 times/week, 3–4 times/week, 5–6 times/week, once/day, 2 times/day, and ≥3 times/day. Information on the portion size was collected only for staple foods (rice, bread, and noodles).

Total physical activity during leisure time was estimated by multiplying the frequency (5 categories from never to ≥5 times/week) and average duration (6 categories from ≤30 min to ≥4 hours) of low, moderate, and powerful intensity exercise. The three exercise levels were summarized and presented as metabolic equivalent (MET)-hours/week. We categorized the study subjects into three groups by smoking status (current, stopped, and never) and drinking status (current, stopped, and never).

Ethanol intake was calculated using the following equation:

$$\begin{array}{rl} \text{Ethanol intake (g/day)} & =\text{frequency (times/day)}\\ & \;\times \text{amount (ml/time)}\\ & \;\times \text{ethanol content (g/ml)} \end{array}$$

Total ethanol intake (g/day) was calculated by summing the ethanol intake from Sake, Syochu, beer, whisky, and wine.

### Anthropometric and biochemical measurements

For the study subjects who attended the Tokushima Prefectural General Health Check-up Center, data on anthropometric measurements (height, weight, and WC), serum TG and GGT were obtained at the time of a routine health check-up. Participants were requested not to eat breakfast, and received a medical check-up between 8:00 AM and 11:30 AM. Height, weight, and WC in centimeters were measured while the participants were dressed in light clothing without shoes. Body mass index (BMI) was calculated as weight in kilograms divided by height in meters squared (kg/m^2^). For the second and third groups, the health check-up was conducted by our research team. The procedure used for the health check-up was almost the same as that of the first group.

### Measurements of nutrient variables

Intake of total energy and 26 nutrients were computed using a program developed by the Department of Public Health, Nagoya City University School of Medicine.^21^^,^^22^ To assess the validity of the FFQ, intakes of total energy and 26 nutrients estimated using the FFQ and 3-day weighed diet records were compared.^22^ The log-transformed and energy-adjusted Pearson’s correlation coefficients ranged from 0.13–0.86 for men and from 0.10–0.66 for women.

We excluded five redundant nutrient variables: total fat; total dietary fiber; polyunsaturated fatty acids; n3-highly unsaturated fatty acids; and energy from alcohol. As a result, 21 nutrients were retained: protein; monounsaturated fatty acids; carbohydrate; soluble dietary fiber; insoluble dietary fiber; saturated fatty acids; n-3 polyunsaturated fatty acids; n-6 polyunsaturated fatty acids; calcium; sodium; potassium; iron; cholesterol; retinol equivalent (EQ); carotene; vitamin D; vitamin E; vitamin B_1_; vitamin B_2_; vitamin C; and folate.

Before factor analysis, we natural log-transformed and adjusted the 21 nutrients for total energy intake. The energy-adjusted nutrient intake was calculated by the following formula: energy-adjusted nutritional intake = crude nutritional intake − (energy intake − mean of energy intake) * *b*, where *b* is a simple regression coefficient between total energy intake (x) and nutrient intake (y).

### Primary outcome

The following formula was used to calculate the FLI^8^:

$$\begin{array}{rl} FLI & =\frac{\left(e^{\left(0.953\times \ln(\text{TG})+0.139\times \text{BMI}+0.718\times \ln(\text{GGT})+0.053\times \text{WC}-15.745\right)}\right)}{\left(1+e^{\left(0.953\times \ln(\text{TG})+0.139\times \text{BMI}+0.718\times \ln(\text{GGT})+0.053\times \text{WC}-15.745\right)}\right)}\\ & \;\times 100 \end{array}$$

The FLI ranges from 0 to 100, and the original recommended cut-offs of the FLI to rule in and rule out fatty liver disease are ≥60 and <30, respectively. In this study, 60 was used as a cut-off level to increase the specificity.

### Statistical analysis

Quantitative data were expressed as medians (with 25^th^ and 75^th^ percentiles), and qualitative data were expressed as numbers and percentages. Wilcoxon’s rank sum test and Chi-square test were used to test for differences in the background characteristics across FLI categories.

We applied factor analysis (principal component) to 21 energy-adjusted nutrients and derived major nutrient patterns. The number of factors retained was based on the following criteria: components with an eigenvalue ≥2.0; a scree plot; and interpretability. The factors identified were rotated by orthogonal transformation (varimax rotation), which maintains uncorrelated factors and produces a simpler structure with greater interpretability. When nutrient patterns were analyzed for each sex separately, extracted factors were similar (eTable 1). Therefore, men and women were combined in the present analysis.

We evaluated the associations of the quartiles of nutrient pattern scores with NAFLD using logistic regression analysis. The first quartile was used as a reference to estimate odds ratios (ORs) and 95% confidence intervals (CIs). In model 1, we adjusted for sex, age, and the three recruitment groups. In model 2, we additionally adjusted for smoking (three categories) and ethanol intake (two categories [≥75 percentile, <75 percentile]), total energy intake (quartiles) and physical activity level (quartiles). The *P* for trend through the quartiles was examined using ordinal categorical variables and a likelihood ratio test. In addition, the associations of nutrient pattern scores and each component of FLI (obesity, high TG, high WC, and high GGT) were examined.

All statistical analyses were performed using the statistical software package SAS version 9.4 (SAS Institute, Cary, NC, USA). A two-sided *P* value of less than 0.05 was considered statistically significant.

### Ethical considerations

The research protocol was reviewed and approved by the ethics committees of Nagoya University Graduate School of Medicine (IRB No. 2010-0939-7), Aichi Cancer Center Research Institute (IRB No. 2016-2-10), Tokushima University Hospital (IRB No. 466-2), and other institutions participating in the J-MICC Study. Written informed consent was obtained from each participant. During the survey, participants were informed that their participation was voluntary, they had the right to withdraw at any point, and data would be confidentially managed.

## RESULTS

Table 1 shows the characteristics of the study subjects according to FLI. Subjects who were defined as having fatty liver (FLI ≥60) were more likely to be men, current or ex-smokers, and physically less active during leisure time. BMI, waist circumference, serum aspartate aminotransferase, alanine aminotransferase, TG, total cholesterol, and GGT were higher, while serum high-density lipoprotein cholesterol was lower, among the subjects with FLI ≥60 than those with FLI <60.

**Table 1.** Background characteristics of the study subjects according to Fatty Liver Index (FLI) category

Characteristics^a^	FLI <60	FLI ≥60	*P*-value^b^
	(*n* = 1,432)	(*n* = 156)	
Age, years	49 (41, 57)	47.5 (40, 57)	0.34
Sex			<0.0001
Male	662 (46.2)	127 (81.4)	
Female	770 (53.8)	29 (18.6)	
Smoking status			<0.0001
Current	225 (15.7)	47 (30.1)	
Stopped	266 (18.6)	53 (34.0)	
Never	941 (65.7)	56 (35.9)	
Alcohol status			0.41
Current	684 (47.8)	83 (53.2)	
Stopped	25 (1.8)	3 (1.9)	
Never	723 (50.5)	70 (44.9)	
Research group			0.02
Occupation	316 (22.1)	33 (21.2)	
Posting	680 (47.5)	91 (58.3)	
Health Check-up	436 (30.5)	32 (20.5)	
Alcohol intake, g/day^c^	0.0 (0, 8.0)	1.2 (0, 9.9)	0.07
Exercise during leisure time, MET-hours/week	3.875 (0.425, 15.75)	2.55 (0.425, 8.725)	0.02
Body mass index, kg/m^2^	22.3 (20.5, 24.3)	28.4 (26.1, 30.8)	<0.0001
Waist circumference, cm	79.5 (72, 85)	95 (90, 101)	<0.0001
Aspartate aminotransferase, IU/L	21 (18, 24)	28 (23, 36)	<0.0001
Alanine aminotransferase, IU/L	17 (13, 24)	39 (28, 54)	<0.0001
Total cholesterol, mg/dL	208.5 (185, 232)	218 (195, 240)	0.0005
Triglycerides, mg/dL	81 (58, 114)	183 (137, 264.5)	<0.0001
High density lipoprotein-cholesterol, mg/dL	60 (51, 70)	47 (42, 53)	<0.0001
γ-Glutamyl transpeptidase, IU/L	21 (15, 31)	51.5 (35, 84)	<0.0001

Nutritional intake			
Energy intake, kcal/day	1,619 (1,452, 1,836)	1,780 (1,547, 2,007)	<0.0001
Protein, g/day	50.2 (44.3, 56.8)	53.2 (45.3, 59.7)	0.02
Monounsaturated fatty acids, g/day	15.6 (13.4, 18.4)	16.2 (13.1, 19.9)	0.60
Carbohydrate, g/day	231.3 (197.5, 272.1)	259.3 (217.9, 307.9)	<0.0001
Soluble dietary fiber, g/day	1.7 (1.4, 2.1)	1.5 (1.2, 1.8)	<0.0001
Insoluble dietary fiber, g/day	6.9 (6.0, 8.4)	6.2 (5.4, 6.9)	<0.0001
Saturated fatty acids, g/day	10.7 (9.1, 12.5)	10.1 (8.5, 11.9)	0.004
n-3 polyunsaturated fatty acids, mg/day	2,084 (1,776, 2,426)	2,128 (1,765, 2,567)	0.76
n-6 polyunsaturated fatty acids, mg/day	10,389 (8,700, 12,249)	10,357 (8,603, 12,794)	0.96
Calcium, mg/day	466 (381, 567)	423 (344, 513)	<0.0001
Sodium, mg/day	1,565 (1,314, 1,826)	1,539 (1,279, 1,877)	0.87
Potassium, mg/day	2,024 (1,773, 2,291)	1,900 (1,670, 2,198)	0.001
Iron, mg/day	6.0 (5.0, 7.2)	5.7 (4.7, 6.8)	0.007
Cholesterol, mg/day	228 (182, 282)	228 (178, 286)	0.88
Retinol equivalent, g/day	701 (541, 991)	558 (506, 955)	<0.0001
Carotene, µg/day	2,717 (2,114, 3,504)	2,114 (1,913, 2,717)	<0.0001
Vitamin D, µg/day	4.9 (4.4, 7.4)	4.9 (4.2, 7.3)	0.23
Vitamin E, mg/day	7.6 (6.6, 9.1)	7.7 (6.3, 8.7)	0.10
Vitamin B1, mg/day	0.62 (0.58, 0.68)	0.63 (0.58, 0.70)	0.17
Vitamin B2, mg/day	0.96 (0.81, 1.13)	0.88 (0.73, 1.11)	0.003
Vitamin C, mg/day	83.2 (65.0, 108.1)	71.3 (53.6, 92.5)	<0.0001
Folate, µg/day	283 (234, 355)	255 (209, 314)	<0.0001

Nutrient pattern scores^d^			
Factor 1	0.03 (0.03)	−0.33 (0.08)	<0.0001
Factor 2	−0.01 (0.03)	0.11 (0.08)	0.16
Factor 3	0.01 (0.03)	−0.10 (0.08)	0.19
Factor 4	−0.01 (0.03)	0.08 (0.08)	0.28

MET, metabolic equivalent.

^a^Median (25%, 75%) or number of subjects (%).

^b^Wilcoxon’s rank sum test or chi-square test.

^c^Participants who drank alcohol above the diagnostic criteria for non-alcoholic fatty liver disease (male >30 g/day; female >20 g/day) were excluded.

^d^The least square means adjusted for sex and standard error.

Table 1 also compares the consumption of total energy and the 21 nutrients according to the FLI categories. Intakes of total energy, protein and carbohydrate were higher, while insoluble and soluble dietary fiber, saturated fatty acids, calcium, potassium, iron, retinol EQ, carotene, vitamin B_2_, C and folate were lower in the high FLI group than in the low FLI group.

Using factor analysis, four nutrient patterns were extracted (Table 2). Based on the correlation coefficients with nutrient intake, Factor 1 was named “vitamins, dietary fiber, iron and potassium pattern”; Factor 2 was named fats and fat-soluble vitamins pattern; Factor 3 was named saturated fat, calcium, vitamin B_2_ and low carbohydrate pattern; and Factor 4 was named sodium, protein and vitamin D pattern. Factor 1 accounted for the largest proportion of the total variance in the nutritional intake, accounting for 28.0%, followed by the second factor (18.9%), the third (16.3%) and the fourth factor (11.2%). The sex-adjusted means and standard errors of Factor 1–4 scores according to the FLI categories are presented in Table 1.

**Table 2.** Factor loading matrix for selected nutrient patterns

Nutrients	Factor 1	Factor 2	Factor 3	Factor 4
Folate	0.91	0.15	0.11	0.10
Carotene	0.86	0.22	0.08	0.08
Insoluble dietary fiber	0.82	0.21	0.18	0.22
Vitamin C	0.79	0.11	0.13	0.15
Iron	0.74	0.13	0.13	0.45
Retinol equivalent	0.73	0.19	0.12	0.06
Soluble dietary fiber	0.72	0.16	0.16	0.41
Potassium	0.70	0.11	0.33	0.21
Monounsaturated fatty acids	0.14	0.94	0.19	0.03
n-3 polyunsaturated fatty acids	0.21	0.87	0.09	0.22
n-6 polyunsaturated fatty acids	0.20	0.86	0.09	0.17
Vitamin E	0.50	0.82	0.12	0.06
Vitamin B_1_	0.05	0.49	0.33	0.40
Cholesterol	0.13	0.36	0.31	0.35
Saturated fatty acids	0.12	0.20	0.92	0.05
Calcium	0.34	−0.05	0.78	0.22
Vitamin B_2_	0.42	0.06	0.75	0.23
Carbohydrate	−0.00	−0.34	−0.80	0.01
Sodium	0.26	0.07	0.04	0.78
Protein	0.19	0.33	0.51	0.65
Vitamin D	0.38	0.20	0.08	0.57

Variance	5.9	4.0	3.4	2.3
Cumulatve variance explained (%)	28.0	46.8	63.2	74.3

The associations between nutrient pattern scores and high FLI are shown in Table 3. Factor 1 scores were inversely and significantly associated with the high FLI category in all models (*P* for trend <0.01). After adjustment for sex, age, and other potential confounders, the OR for the highest quartile group relative to the lowest quartile group was 0.41 (95% CI, 0.22–0.74). Factor 2 scores were marginally significantly associated with high FLI (*P* for trend = 0.07). There were no significant relationships between Factor 3 or 4 scores and the high FLI category.

**Table 3.** Associations of four factor scores with high Fatty Liver Index (FLI)

FLI ≥60	Q1	Q2	Q3	Q4	*P* for trend
	OR	OR (95% CI)	OR (95% CI)	OR (95% CI)	
Factor 1	*n* = 397	*n* = 397	*n* = 397	*n* = 397	
Number of cases (%)	66 (16.6)	41 (10.3)	31 (7.8)	18 (4.5)	
Model 1^a^	1.00	0.66 (0.43–1.01)	0.53 (0.33–0.84)	0.36 (0.20–0.63)	0.0002
Model 2^b^	1.00	0.70 (0.45–1.08)	0.55 (0.34–0.89)	0.41 (0.22–0.74)	0.001

Factor 2	*n* = 397	*n* = 397	*n* = 397	*n* = 397	
Number of cases (%)	35 (8.8)	39 (9.8)	33 (8.3)	49 (12.3)	
Model 1	1.00	1.31 (0.80–2.15)	1.18 (0.71–1.98)	1.70 (1.05–2.77)	0.05
Model 2	1.00	1.27 (0.77–2.10)	1.17 (0.69–1.98)	1.63 (1.00–2.69)	0.07

Factor 3	*n* = 397	*n* = 397	*n* = 397	*n* = 397	
Number of cases (%)	56 (14.1)	42 (10.6)	25 (6.3)	33 (8.3)	
Model 1	1.00	0.80 (0.51–1.24)	0.51 (0.31–0.84)	0.80 (0.49–1.28)	0.11
Model 2	1.00	0.83 (0.52–1.29)	0.54 (0.32–0.90)	0.86 (0.53–1.40)	0.22

Factor 4	*n* = 397	*n* = 397	*n* = 397	*n* = 397	
Number of cases (%)	36 (9.1)	40 (10.1)	36 (9.1)	44 (11.1)	
Model 1	1.00	1.16 (0.72–1.89)	1.07 (0.65–1.75)	1.39 (0.86–2.25)	0.24
Model 2	1.00	1.17 (0.71–1.91)	1.02 (0.62–1.70)	1.42 (0.87–2.32)	0.24

CI, confidence interval; OR, odds ratio; Q, quartile.

^a^Adjusted for sex, age, and research group.

^b^Additionally adjusted for smoking, amount of ethanol intake, total energy intake, and physical activity level.

Table 4 shows the associations of nutrient pattern scores with each component of FLI. There were significant inverse associations between Factor 1 scores and high BMI and high WC, and between Factor 3 scores and high WC. On the other hand, Factor 2 scores were positively and significantly associated with high BMI and high WC.

**Table 4.** Associations of four factor scores with each Fatty Liver Index component

	Q1	Q2	Q3	Q4	*P* for trend
	OR	OR (95% CI)	OR (95% CI)	OR (95% CI)	
Body mass index ≥25 kg/m^2^					
Factor 1	*n* = 397	*n* = 397	*n* = 397	*n* = 397	
Number of cases (%)	127 (32.0)	108 (27.2)	92 (23.2)	62 (15.6)	
Model 2^a^	1.00	0.92 (0.66–1.26)	0.72 (0.52–1.01)	0.48 (0.33–0.71)	0.0002
Factor 2	*n* = 397	*n* = 397	*n* = 397	*n* = 397	
Number of cases (%)	88 (22.2)	105 (26.5)	82 (20.7)	114 (28.7)	
Model 2	1.00	1.45 (1.03–2.04)	1.10 (0.77–1.57)	1.64 (1.17–2.32)	0.03
Factor 3	*n* = 397	*n* = 397	*n* = 397	*n* = 397	
Number of cases (%)	118 (29.7)	108 (27.2)	83 (20.9)	80 (20.2)	
Model 2	1.00	0.98 (0.71–1.36)	0.75 (0.53–1.05)	0.77 (0.54–1.09)	0.06
Factor 4	*n* = 397	*n* = 397	*n* = 397	*n* = 397	
Number of cases (%)	96 (24.2)	99 (24.9)	101 (25.4)	93 (23.4)	
Model 2	1.00	1.05 (0.75–1.47)	1.09 (0.78–1.52)	0.99 (0.70–1.39)	0.97

Triglycerides ≥150 mg/dL					
Factor 1	*n* = 397	*n* = 397	*n* = 397	*n* = 397	
Number of cases (%)	84 (21.2)	64 (16.1)	69 (17.4)	53 (13.4)	
Model 2	1.00	0.83 (0.57–1.21)	1.00 (0.68–1.47)	0.89 (0.58–1.38)	0.82
Factor 2	*n* = 397	*n* = 397	*n* = 397	*n* = 397	
Number of cases (%)	81 (20.4)	67 (16.9)	66 (16.6)	56 (14.1)	
Model 2	1.00	0.90 (0.61–1.31)	1.00 (0.68–1.46)	0.73 (0.49–1.09)	0.20
Factor 3	*n* = 397	*n* = 397	*n* = 397	*n* = 397	
Number of cases (%)	84 (21.2)	68 (17.1)	61 (15.4)	57 (14.4)	
Model 2	1.00	0.90 (0.62–1.31)	0.89 (0.60–1.30)	0.95 (0.64–1.41)	0.74
Factor 4	*n* = 397	*n* = 397	*n* = 397	*n* = 397	
Number of cases (%)	62 (15.6)	72 (18.1)	70 (17.6)	66 (16.6)	
Model 2	1.00	1.26 (0.86–1.86)	1.19 (0.81–1.77)	1.13 (0.76–1.68)	0.63

Waist circumference (male ≥90 cm, female ≥80 cm)					
Factor 1	*n* = 397	*n* = 397	*n* = 397	*n* = 397	
Number of cases (%)	120 (30.2)	111 (28.0)	115 (29.0)	110 (27.7)	
Model 2	1.00	0.81 (0.59–1.12)	0.79 (0.57–1.10)	0.63 (0.44–0.90)	0.02
Factor 2	*n* = 397	*n* = 397	*n* = 397	*n* = 397	
Number of cases (%)	104 (26.2)	105 (26.5)	115 (29.0)	132 (33.3)	
Model 2	1.00	1.06 (0.77–1.47)	1.21 (0.88–1.68)	1.61 (1.16–2.23)	0.003
Factor 3	*n* = 397	*n* = 397	*n* = 397	*n* = 397	
Number of cases (%)	127 (32.0)	110 (27.7)	114 (28.7)	105 (26.5)	
Model 2	1.00	0.81 (0.59–1.12)	0.85 (0.62–1.17)	0.69 (0.50–0.96)	0.04
Factor 4	*n* = 397	*n* = 397	*n* = 397	*n* = 397	
Number of cases (%)	99 (24.9)	104 (26.2)	131 (33.0)	122 (30.7)	
Model 2	1.00	1.09 (0.79–1.52)	1.43 (1.04–1.96)	1.28 (0.93–1.77)	0.05

γ-Glutamyl transpeptidase (male ≥85 IU/L, female ≥38 IU/L)					
Factor 1	*n* = 397	*n* = 397	*n* = 397	*n* = 397	
Number of cases (%)	34 (8.6)	37 (9.3)	43 (10.8)	37 (9.3)	
Model 2	1.00	1.04 (0.63–1.72)	1.16 (0.71–1.91)	0.91 (0.53–1.58)	0.85
Factor 2	*n* = 397	*n* = 397	*n* = 397	*n* = 397	
Number of cases (%)	32 (8.1)	39 (9.8)	46 (11.6)	34 (8.6)	
Model 2	1.00	1.34 (0.82–2.21)	1.64 (1.01–2.68)	1.20 (0.71–2.03)	0.36
Factor 3	*n* = 397	*n* = 397	*n* = 397	*n* = 397	
Number of cases (%)	44 (11.1)	38 (9.6)	33 (8.3)	36 (9.1)	
Model 2	1.00	0.86 (0.54–1.37)	0.70 (0.43–1.14)	0.79 (0.48–1.28)	0.24
Factor 4	*n* = 397	*n* = 397	*n* = 397	*n* = 397	
Number of cases (%)	32 (8.1)	32 (8.1)	44 (11.1)	43 (10.8)	
Model 2	1.00	1.01 (0.60–1.68)	1.38 (0.86–2.26)	1.28 (0.78–2.09)	0.19

CI, confidence interval; OR, odds ratio; Q, quartile.

^a^Adjusted for sex, age, research group, smoking, amount of ethanol intake, total energy intake, and physical activity level.

## DISCUSSION

The present study showed a significant inverse association between Factor 1 scores and the high FLI category. Additional analyses for each component of FLI showed that Factor 1 scores were associated with lower BMI and WC, suggesting that obesity and abdominal obesity were intermediate variables between Factor 1 scores and NAFLD. Furthermore, nutrients having strong positive correlations with Factor 1 scores (potassium, carotene, folate, vitamin C, soluble and insoluble dietary fiber) were associated with a lower prevalence of high FLI (data not shown). Dietary fiber, especially when consumed as whole foods, reduces energy density in foods and increases satiety, thus leading to prevention of obesity in the long term.^26^ Insoluble dietary fiber is known to reduce insulin resistance,^27^ which may reduce free fatty acid fluxes from the adipose tissue to the liver.^28^ In addition, vitamins are known to be involved in the pathogenesis of NAFLD in various ways. For instance, vitamin A plays an important role in hepatic fatty acid β oxidation in nonalcoholic steatohepatitis animal model, and vitamin C protects hepatic cells from lipotoxicity-induced oxidative stress.^29^ Since the nutrients positively correlated with Factor 1 score are abundantly contained in vegetables and fruits, the present results are in line with previous studies using diet pattern analysis. For instance, cross-sectional studies in China reported that dietary patterns characterized by high intake of whole grains, tubers, vegetables, and beans, were associated with a lower prevalence of NAFLD.^12^^,^^13^ In an elderly population in Brazil, a healthy diet pattern with high intake of fruits, greens and vegetables, olive oil, margarine, bread and white meats was negatively associated with NAFLD.^25^

In previous studies, a Western/high fat diet pattern was positively associated with NAFLD^15^ and a closely related condition, metabolic syndrome.^30^ The fats and fat-soluble vitamins pattern, which resembled the high fat diet pattern, was extracted in our previous study using approximately 30,000 subjects from seven study cites of the J-MICC study, and was positively associated with the prevalence of metabolic syndrome.^31^ In the present study, Factor 2 scores were significantly associated with high BMI and high WC, and marginally significantly associated with high FLI (*P* = 0.07). It is known that high fat diet has high energy density, so it may lead to excessive caloric intake and obesity in the long term. On the other hand, Factor 2 scores had no associations with high serum TG or GGT. This may be because of the high factor loadings for some beneficial nutrients, such as n3-polyunsaturated fatty acids and vitamin E. Eicosapentaenoic acid and docosaexaenoic acid reduce serum levels of triglycerides, and have anti-inflammatory effects.^32^ In addition, vitamin E showed anti-steatotic, anti-inflammatory, and anti-fibrotic effects in experimental NAFLD animals.^29^

Factor 3 scores (saturated fat, calcium, vitamin B_2_, and low carbohydrate pattern) were associated with lower BMI and WC, but not with other components of FLI or high FLI. One possible explanation for this result is the negative correlation of Factor 3 scores with carbohydrate intake (*r* = −0.80). Low carbohydrate diet is known to be effective for weight reduction.^33^ In addition, although the interpretation of Factor 3 was not straightforward, examination of the correlation with food/food groups revealed a close positive correlation of this factor with the frequency of milk intake (Spearman *r* = 0.82). It is known that consumption of calcium or dairy products impairs intestinal absorption of fat by the formation of insoluble soap, leading to increased fecal fat excretion and loss of body fat.^34^ The possible reasons for the lack of a significant inverse association of Factor 3 scores with NAFLD are insufficient statistical power because of the low prevalence of NAFLD, and the random misclassification of NAFLD status due to no image diagnosis. When 30 was used as a cut-off point of FLI, Factor 3 scores were significantly and inversely associated with high FLI (*P* for trend = 0.004, eTable 2). In addition, when multiple linear regression was used, higher Factor 3 scores were significantly associated with lower FLI (*P* = 0.01, data not shown). Regarding dairy product intake and NAFLD, one cross-sectional study in China showed an inverse association between yogurt consumption and lower prevalence of NAFLD.^35^ However, in a recent meta-analysis, no significant association was found between dairy product intake and NAFLD.^36^

The advantage of the present study is that it is the first study to examine the association between nutrient patterns and NAFLD. In addition, by using this approach, we could handle closely correlated nutrients and avoid the problem of collinearity. On the other hand, this study also has several limitations. First, because this was a cross-sectional study, the time sequence of nutrient intake and onset of NAFLD is unclear. Second, this study did not use image diagnosis or liver biopsy, the gold standard for the diagnosis of NAFLD. Third, although this study used a validated FFQ, measurement error in the assessment of nutrient intake may have been unavoidable. Fourth, although several life-style factors were adjusted as potential confounders, the possibility of residual confounding or confounding by unknown factors remains. Fifth, because we used an “a posteriori approach” and extracted nutrient patterns from the data at hand, the derived nutrient patterns may be unique to the study population and may not be directly applicable to other populations. However, at least two extracted factors were related to well-known dietary patterns (ie, healthy/prudent and Western/high fat patterns).^30^ Sixth, some nutrient patterns extracted in the present study were strongly influenced by food groups or a food. For instance, Factor 3 scores were highly correlated with milk intake. Therefore, it was easier to interpret the association between Factor 3 and NAFLD in terms of food intake, rather than combination of nutrients. Finally, since almost all study subjects were Japanese, the results may not be directly generalizable to other ethnic groups.

In summary, the present results suggest that a nutrient pattern rich in vitamins, fiber, iron, and potassium was associated with lower prevalence of NAFLD in a Japanese population. Obesity and abdominal obesity may be intermediate variables for the association between this nutrient pattern and NAFLD.

## SUPPLEMENTARY MATERIAL

eTable 1. Factor loading matrix for selected nutrient patterns by sex

eTable 2. Associations of four factor scores with high Fatty Liver Index (Cut-off level = 30)
