# Supplementary material for: Association Between Nutrient Patterns and Fatty Liver Index: Baseline Survey of the Japan Multi-Institutional Collaborative Cohort Study in Tokushima, Japan
Source: J Epidemiol. 2022 Aug 5;32(8):376–83. doi: 10.2188/jea.JE20200447 (PMC9263617; doi:10.2188/jea.JE20200447)
Supplement: Supplementary file 1 [file je-32-376-s001.pdf]

eTable 1. Factor loading matrix for selected nutrient patterns by sex

Nutrients	Male				Female			
	Factor 1	Factor 2	Factor 3	Factor 4	Factor 1	Factor 2	Factor 3	Factor 4
Folate	0.91	0.14	0.13	0.04	0.90	0.14	0.08	0.10
Carotene	0.85	0.20	0.05	0.07	0.86	0.22	0.04	0.07
Vitamin C	0.82	0.09	0.10	0.13	0.76	0.10	0.12	0.14
Insoluble dietary fiber	0.79	0.23	0.16	0.25	0.84	0.18	0.12	0.20
Iron	0.72	0.14	0.13	0.46	0.75	0.12	0.14	0.42
Retinol equivalent	0.70	0.18	0.17	-0.04	0.75	0.20	0.08	0.08
Soluble dietary fiber	0.66	0.18	0.15	0.52	0.77	0.12	0.10	0.35
Potassium	0.62	0.08	0.34	0.24	0.74	0.12	0.32	0.18
Vitamin D	0.45	0.19	0.19	0.39	0.35	0.23	0.04	0.63
Monounsaturated fatty acids	0.12	0.95	0.16	0.04	0.13	0.93	0.19	-0.03
n-3 polyunsaturated fatty acids	0.26	0.87	0.13	0.15	0.18	0.88	0.05	0.22
n-6 polyunsaturated fatty acids	0.20	0.86	0.08	0.20	0.16	0.85	0.06	0.13
Vitamin E	0.46	0.84	0.12	0.07	0.52	0.80	0.08	0.00
Vitamin B_1	0.02	0.50	0.32	0.42	0.07	0.51	0.32	0.34
Cholesterol	0.07	0.38	0.30	0.36	0.15	0.36	0.30	0.27
Saturated fatty acids	0.07	0.24	0.90	0.06	0.07	0.17	0.93	-0.01
Calcium	0.30	-0.07	0.80	0.20	0.32	-0.06	0.78	0.21
Vitamin B_2	0.41	0.06	0.75	0.18	0.39	0.04	0.77	0.21
Carbohydrate	-0.06	-0.41	-0.74	-0.02	0.08	-0.30	-0.82	0.06
Sodium	0.24	0.11	0.07	0.85	0.28	0.03	0.02	0.76
Protein	0.23	0.36	0.55	0.59	0.15	0.32	0.50	0.65
Variance	5.6	4.1	3.4	2.3	6.0	3.9	3.4	2.2
Cumulatve variance explained (%)	26.6	46.3	62.4	73.3	28.5	46.9	63.3	73.5

eTable 2. Associations of four factor scores with high Fatty Liver Index (Cut-off level=30)

FLI ≥30	Q1<br>OR	Q2<br>OR (95% CI)	Q3<br>OR (95% CI)	Q4<br>OR (95% CI)	*P* for trend [c]
Factor 1	n=397	n=397	n=397	n=397	
Number of cases (%)	153 (38.5)	122 (30.7)	105 (26.5)	80 (20.2)	
Model 1 [a]	1.00	0.84 (0.61-1.16)	0.71 (0.51-0.98)	0.59 (0.41-0.86)	0.003
Model 2 [b]	1.00	0.87 (0.63-1.20)	0.71 (0.51-1.00)	0.62 (0.42-0.91)	0.008
Factor 2	n=397	n=397	n=397	n=397	
Number of cases (%)	127 (32.0)	112 (28.2)	98 (24.7)	123 (31.0)	
Model 1	1.00	1.02 (0.73-1.43)	0.96 (0.68-1.35)	1.28 (0.92-1.79)	0.21
Model 2	1.00	1.01 (0.72-1.41)	0.94 (0.66-1.33)	1.22 (0.87-1.72)	0.33
Factor 3	n=397	n=397	n=397	n=397	
Number of cases (%)	157 (39.6)	121 (30.5)	94 (23.7)	88 (22.2)	
Model 1	1.00	0.74 (0.54-1.02)	0.59 (0.42-0.82)	0.64 (0.46-0.90)	0.003
Model 2	1.00	0.75 (0.54-1.04)	0.59 (0.42-0.83)	0.65 (0.46-0.92)	0.004
Factor 4	n=397	n=397	n=397	n=397	
Number of cases (%)	110 (27.7)	114 (28.7)	124 (31.2)	112 (28.2)	
Model 1	1.00	1.11 (0.80-1.56)	1.31 (0.94-1.83)	1.08 (0.77–1.52)	0.46
Model 2	1.00	1.12 (0.80-1.57)	1.31 (0.94-1.84)	1.10 (0.78-1.56)	0.40

CI, confidence interval; OR, odds ratio; Q, quartile.

[a] Adjusted for sex, age, and research group.

[b] Additionally adjusted for smoking, amount of ethanol intake, total energy intake, and physical activity level.

[c] *P* for trend was calculated using Wald test.
